# The Microcirculation Is Unchanged in Neonates with Severe Respiratory Failure after the Initiation of ECMO Treatment

**DOI:** 10.1155/2012/372956

**Published:** 2012-05-23

**Authors:** Anke P. C. Top, Erik A. B. Buijs, Patrick H. M. Schouwenberg, Monique van Dijk, Dick Tibboel, Can Ince

**Affiliations:** ^1^Intensive Care, Erasmus Medical Center-Sophia Children's Hospital, University Medical Center, P.O. Box 2060, 3000 CB, Rotterdam, The Netherlands; ^2^Pediatric Intensive Care Unit, Addenbrooke's Hospital, Cambridge CB2 0QQ, UK; ^3^Department of Intensive Care, Erasmus Medical Center, University Medical Center, P.O. Box 2040, 3000 CA, Rotterdam, The Netherlands

## Abstract

*Purpose*. Venoarterial extracorporeal membrane oxygenation (VA-ECMO) is known to improve cardiorespiratory function and outcome in neonates with severe respiratory failure. We tested the hypothesis that VA-ECMO therapy improves the microcirculation in neonates with severe respiratory failure. *Methods*. This single-center prospective observational pilot study took place in an intensive care unit of a level III university children's hospital. Twenty-one-term neonates, who received VA-ECMO treatment, were included. The microcirculation was assessed in the buccal mucosa, using Orthogonal Polarization Spectral imaging, within 24 hours before (T1) and within the first 24 hours after initiation of ECMO treatment (T2). Data were compared to data of a ventilated control group (*N* = 7). *Results*. At baseline (T1), median functional capillary density (FCD), microvascular flow index (MFI), and heterogeneity index (HI) did not differ between the ECMO group and the control group. At T2 the median FCD was lower in the control group (median [range]: 2.4 [1.4–4.2] versus 4.3 [2.8–7.4] cm/cm^2^; *P* value <0.001). For MFI and HI there were no differences at T2 between the two groups. *Conclusion*. The perfusion of the microcirculation does not change after initiation of VA-ECMO treatment in neonates with severe respiratory failure.

## 1. Introduction

Extracorporeal membrane oxygenation (ECMO) is a cardiopulmonary bypass technique used as life support in selected newborns and children with acute reversible cardiorespiratory failure when conventional management is not successful [1, 2]. Worldwide, over 24,000 neonates have been treated with ECMO for respiratory problems [1–3].

ECMO therapy gives time to restore normal pulmonary oxygenation in neonates with severe respiratory failure who do not respond to maximal conventional therapy and is regarded as a bridge to recovery [1, 2, 4]. The institution of venoarterial ECMO (VA-ECMO) partly takes over oxygenation, and carbon dioxide removal and thereby allows ventilator settings to be reduced and restores circulation [4].

The institution of an ECMO circuit in neonates results in an expansion of the circulating volume by approximately factor 2.5. In VA-ECMO, the heart is bypassed and flow in the systemic circulation is generated mostly by the ECMO pump, producing nonpulsatile flow. Especially during high ECMO flow rate (120–200 mL/kg/min), this results in disturbance of the physiologic blood flow, which can be represented by a flattening of the arterial pulse waves on invasive blood pressure monitoring [4, 5].

In neonatal patients with severe respiratory failure, who meet the criteria for ECMO treatment [4], the circulation and oxygenation are severely compromised. Reflecting this condition, these patients' microcirculatory parameters are significantly reduced before VA-ECMO [6]. At the time when the patient no longer needs ECMO, the microcirculatory parameters are improved, correlating well with an improvement in clinical condition [6]. After VA-ECMO initiation, circulation and oxygenation generally improve rapidly and patients show a decrease in the need for vasoactive medication. Direct effects of artificial, nonpulsatile ECMO flow on the microcirculation are still not completely understood.

Based on clinical observations and the instant decrease of need for vasoactive medication after the start of ECMO therapy, we hypothesize that microcirculatory alterations observed in neonates with severe respiratory failure improve with the initiation of ECMO therapy.

## 2. Materials and Methods

### 2.1. Patients

Neonatal patients (aged ≤28 days) admitted to our intensive care unit and treated with VA-ECMO were enrolled in this study. Patients were treated with ECMO, according to our unit specific policy. Patients suffering from congenital heart disease were excluded.

In accordance with the guidelines of the medical ethical review board of our hospital, informed consent was waived when standard therapy is monitored by noninvasive techniques.

Patients in the study group had severe cardiorespiratory failure and hypoxemia despite adequate conventional treatments such as mechanical ventilation, sedation, muscle paralysis, vasoactive drugs, and nitric oxide inhalation. All patients met the established entry criteria for ECMO [4]. Starting ECMO treatment in a newborn implies a massive increase of the circulating volume (the priming volume of the used system is ±350 mL, which is about 1.5 times the circulating volume of a newborn baby). The ECMO system was primed with a combination of Ringer's lactate, packed red blood cells and albumen. Bicarbonate and calcium were added based on bloodgas analysis of the priming fluid. Initially the aimed ECMO flow rate was 150–200 mL/kg/min and after 24 hours weaning of the flow was started under guidance of changes in arterial pO_2_ and signs of pulmonary hypertension.

In addition to the microvascular measurements, patient's demographic and clinical parameters, such as gender, birth weight, gestational age, postnatal age, diagnosis, ECMO flow, heart rate, blood pressure, mean arterial blood pressure, body temperature, administered medication, hemoglobin, and hematocrit levels were recorded. Data were compared to data of control subjects, with severe respiratory failure, who did not receive ECMO treatment. In the control group, patients were measured several consecutive days after admission. The first two measurements on consecutive days were taken to serve as control for T1 and T2 and to evaluate the changes without ECMO treatment.

### 2.2. Procedures

The microcirculation was assessed within 24 hours before start of ECMO (T1) and within 24 hours after start of ECMO (T2). OPS imaging [7] was used to visualize the microvascular network of the buccal mucosa. The measurements were done with a CYTOSCAN E-II Backfocus-type device (Cytometrics, Philadelphia, PA, USA), using the 5x objective.

Before the measurements, saliva was gently removed with gauze. The lens of the OPS-imaging device was covered with a disposable sterile cap and was applied to the buccal mucosa without pressure, as described before [6]. Images from 3 different regions were obtained and stored on digital videotapes, using a Sony DSR-20P digital video recorder. Segments of 5 seconds were selected and captured in AVI (audio video interleave) format. Video segments that did not meet quality criteria were discarded [6, 8]. For every measurement, the functional capillary density (FCD), microvascular flow index (MFI), and heterogeneity index (HI) of the different video segments were averaged. If only one segment met the quality criteria, this score was taken. (This was the case for 2 ECMO patients at T2 and 1 control patient at T1).

### 2.3. Microcirculatory Analysis

Quantification of the images was performed as described previously [6, 7]. To investigate vessel density, the images were analyzed with the Capiscope software program (version 3.7.1.0, KK Technology 1993–2000). For the FCD calculation, the analyst is required to trace out the path of the moving red blood cells within the capillaries (vessels, smaller than 10 *μ*m). A functional capillary is defined as a capillary that has at least one red blood cell moving through it, during the observation period. Dividing the length of the perfused capillaries by the area gives the functional capillary density value expressed in cm/cm^2^.

The flow pattern was studied using the MFI, and the HI [8]. For MFI the predominant type of flow for small, medium, and large vessels in every quadrant of the images was determined, as described before by Boerma et al. [9]. For every measurement, the scores for the different video segments were averaged. If only one segment met the quality criteria, this score was taken. HI was calculated as the highest site flow velocity minus the lowest site flow velocity, divided by the mean flow velocity of all sites per measurement [8].

### 2.4. Statistical Analysis

The data were analyzed using SPSS 17.0. Continuous data are presented as median and range, discrete data as number and percentage. The intergroup differences at T1 were assessed using the Mann Whitney test. Changes over time were assessed using analysis of covariance (ANCOVA) with the T2 measurement as outcome variable, the groups as factor, and the T1 measurement as covariate. In this way, differences at T2 are corrected for the baseline measurements. The level of significance was set at *P* < 0.05.

## 3. Results

During the study period, 31 VA-ECMO patients were eligible for inclusion. Twenty-one patients were included in the study. Four patients were missed for inclusion due to logistic reasons (a researcher was not contacted in time or no investigator or camera available). Six patients were excluded because their video segments did not meet the quality criteria [6]. The excluded ECMO patients did not differ from the included ECMO patient group for gestational age, postnatal age, diagnosis, duration of ECMO treatment, or mortality. In the control group, four patients were missed for inclusion and seven patients had to be excluded due to insufficient quality of the images. Demographic data are presented in Table 1, clinical data in Table 2, and microcirculatory data obtained by SDF are presented in Table 3.

**Figure 1:**
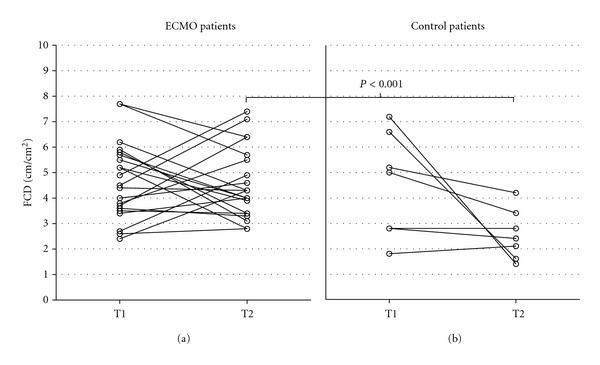
Diagram showing the functional capillary density (FCD). (a): ECMO patients, (b): ventilated control patients. No difference in median FCD was seen at T1 between the two groups: 4.5 cm/cm^2^ (range 2.4–7.7) versus 5.0 cm/cm^2^ (range 1.8–7.2), *P* value = 0.811. At T2, FCD was higher in ECMO group than in the control group: 4.3 cm/cm^2^ (range 2.8–7.7) versus 2.4 cm/cm^2^ (range 1.4–4.2), *P* value <0.001.

At baseline (T1), median FCD did not differ between the ECMO group and the control group (median [range]: 4.5 [2.4–7.7] versus 5.0 [1.8–7.2] cm/cm^2^, *P* value = 0.811) (Figure 1). ANCOVA analysis indicated that at T2 the median FCD was 1.9 cm/cm^2^ lower in the control group than it was in the ECMO group (median [range]: 2.4 [1.4–4.2] versus 4.3 [2.8–7.4] cm/cm^2^; *P* value <0.001). For MFI and HI, there was neither a difference at T1 nor a difference at T2 between the two groups (see Table 3 for absolute MFI values and HI values per vessel type as well as the associated *P* values).

 At baseline, the disease severity indices oxygenation index (median [range]: 31 [5–94] versus 5 [3–13]; *P* value = 0.004) and the PELOD score (median [range]: 20 [11–31] versus 11 [11–20]; *P* value = 0.006) were more unfavourable for the ECMO patients than for the control patients. The heart rate was higher in the ECMO patients (median [range]: 180 [120–220] versus 138 [113–191] bpm; *P* value = 0.046), whereas the mean arterial blood pressure and the pulse pressure did not differ. The need for vasoactive medication as indicated by the vasopressor score did not differ between the two groups at T1. Mean airway pressure (median [range]: 18 [12–27] versus 14 [9–16] cm H_2_O; *P* value = 0.019) and the median dosage of inhaled nitric oxide (median [range]: 20 [0–40] versus 0 [0–19] ppm; *P* value = 0.012) were both higher in the ECMO patients than in the control patients.

 At T2, ANCOVA analysis indicated that there was no difference in OI between the ECMO group and the control group. The heart rate and the mean arterial blood pressure did not differ. Pulse pressure was lower in the ECMO patients than in the control patients (median [range]: 10 [0–33] versus 24 [15–32]; *P* value <0.001). The vasopressor score did not differ at T2, nor did the mean airway pressure. Regarding the dosage of inhaled nitric oxide, ANCOVA analysis indicated that the need for more inhaled nitric oxide in the ECMO patients at T1 had disappeared at T2.

All patients in the control group survived. Three patients in the ECMO-treated group (2 diagnosed with CDH, 1 with CCAM) did not survive, due to recurrent and therapy-resistant pulmonary hypertension. Subanalysis showed that neither FCD nor MFI, nor HI differed between the ECMO survivors and the ECMO nonsurvivors at T1 and at T2.

## 4. Discussion

The main finding of this study was that there was no change in microcirculatory parameters after the start of VA-ECMO therapy in patients with severe respiratory failure. In both the ECMO and the control group, the FCD at T1 was significantly lower than FCD values of neonates without any respiratory or cardiovascular problems (who served as a control group in a previous study [6]). The FCD in those patients was 8.1 cm/cm^2^ (range, 6.6–9.4). MFI values in both study groups were relatively high and HI values relatively low, in contrast to observations in patients with sepsis. There was no difference in MFI and HI between the two groups at T1 and T2. Deterioration of the FCD was observed in patients with severe respiratory failure, who did not receive ECMO treatment. Despite the fact that patients in the ECMO group were more severely ill, in comparison to the patients in the ventilated control group (Oxygenation Index and PELOD score in ECMO group significantly higher), ECMO succeeded to better microcirculatory support compared to solely conservative treatment with mechanical ventilation and pharmacologic support.

Thus, ECMO seems to prevent a further deterioration of microcirculatory perfusion. The start of ECMO instigates an instant improvement in oxygenation, which makes vasopressors and the use of high mean airway pressures instantly redundant. No correlation between the vasopressor score or the main airway pressure and FCD was found.

Deterioration of microvascular perfusion in patients in the ventilated control group was not correlated with mortality. This is in contrast with observations in patients with severe sepsis [10–12]. The underlying pathophysiology in patients in our study is different from sepsis. Therefore, data from patients with sepsis cannot be extrapolated to this patient group. Both patient groups revealed a relatively normal flow pattern and selectively affected vessel density. At this stage, it is not clear if this could be explained by their specific hemodynamic pattern. Patients in this study suffered from hypoxic respiratory failure, mainly due to failure of adequate feto-neonatal transition of the circulation. Typically, these patients display a hemodynamic pattern with persistent pulmonary hypertension of the neonate (PPHN), which is clinically characterized by a persistent high pulmonary vascular resistance and an abnormal vascular response, leading to worsening of gas exchange and shunting (intracardiac, extracardial, and intrapulmonary) and right ventricular failure. PPHN occurs as a primary disease or in association with abnormal lung development, for example, in congenital diaphragmatic hernia and is a critical determinant of morbidity and mortality [13].

All patients had pulmonary hypertension, assessed by echocardiography and differences in the pre- and postductal oxygen saturation (due to shunting through persistent fetal pathways such as the ductus arteriosus). This can compromise the pulmonary venous return and preload of the left ventricle and, therefore, influence global hemodynamics. No measures of cardiac output (CO) were available in this study, so this cannot be verified.

During cardiopulmonary bypass (CPB) in adults, microcirculatory alterations have been described before [14–17]. We found one report on microcirculatory alterations during CPB in neonates where OPS was used, which shows a reduction in vessel density during CPB [18].

The circulatory volume increases by about 150%, when a newborn is attached to an ECMO circuit. Therefore, it is necessary that the system is primed with blood products. The addition of these products is titrated against normal values for the age. Thus, with ECMO, blood is transfused, which could improve the microcirculation [19]. However, there was no increment in the hemoglobin level, to support this. With the attachment of the system, a large amount of fluid is administered, which could influence the perfusion of the microcirculation [20]. Due to the relatively large amount of circulating volume in the system, it is difficult to comment on volume expansion in the patient in absolute numbers. During cannulation and shortly afterwards extra fluid was administered on discretion of the treating physician, based on clinical judgment and following standard unit policies and procedures.

 Disturbance of physiologic flow also triggers the catecholamine system leading to vasoconstriction and altered tissue perfusion [21]. Although the mechanism behind this is not completely understood, Agati et al. [22–24] reported that in cardiac patients on CPB nonpulsatile flow seemed to affect the microcirculation and organ perfusion in a more negative way than pulsatile flow did. No correlation between ECMO flow and FCD was seen in our study.

All in all, the initiation of ECMO therapy instigates many changes in the homeostasis of the critically ill patient. It is difficult to unravel the complex processes that take place and to assess separate factors, in order to understand the effect of the different components of the treatment. Nowadays, the importance of microcirculatory improvement is recognized [25, 26]. With this paper, we have shown that the current way of using ECMO treatment stabilizes the microcirculation, but does not restore microvascular density. More research is needed to explore the different factors that have influence on the microcirculation. In addition, follow-up investigations of the microcirculation are necessary as well as comparison of survivors and nonsurvivors within the group that received ECMO treatment. In this way, the prognostic value of microcirculatory parameters can be determined.

There were some limitations to our study. First, the lack of CO measurements limits the possibility to relate microvascular observations to global hemodynamics. Changes in CO could possibly play a role in the decrease of FCD between T1 and T2 in the control group. In children, mixed venous saturation and cardiac output are not routinely measured. A prerequisite for adequate CO monitoring is a tool that is accurate, is easy to use, and has an acceptable risk-benefit profile. These three factors have constituted the major hurdle to bedside pediatric cardiac output measurement to date [27]. The reliability of echocardiography evaluation of cardiac output in children is debatable because even in the hands of experienced operators the inter- and intraindividual variation is large [28].

Second, the control group consisted entirely of patients with CDH, while the ECMO group also contained patients with severe respiratory failure and pulmonary hypertension due to other causes. Patients with CDH suffer from a specific hemodynamic pattern, based on a structural congenital abnormality [13]. This could possibly have different implications on the development of global hemodynamics and the microcirculation.

Unfortunately, the exact amounts of priming fluids and fluids, given during or shortly after the cannulation procedure prior to T2, are not well documented. In addition, 12 of the 21 ECMO patients were first measured within 2 hours of IC admission. In these patients, no reliable data on the amount of fluid administration prior to admission was available. Therefore, we are unable to provide reliable data for fluid balance, fluid amount, and type of fluids administered for ECMO patients in this study.

In this pilot study, the microcirculation was assessed before and after the start of ECMO; therefore, long-term effects of ECMO could not be evaluated. In addition, the median time interval for the subsequent SDF measurements in the ECMO group was shorter than that of the control group. The earlier microcirculatory evaluation in the ECMO group might be of influence on our results.

Finally, this study is observational and not randomized controlled, which skews outcome data. If children in the control group had disposed progressive respiratory and/or circulatory failure, they would have received ECMO treatment. From an ethical perspective, randomization for this type of treatments is unacceptable.

## 5. Conclusion

The perfusion of the microcirculation does not change after initiation of VA-ECMO treatment in neonates with severe respiratory failure.

## Figures and Tables

**Table 1:** Demographic data.

		ECMO *N* = 21	Controls *N* = 7
Gestational age [weeks]		39.0 (34.4–42.5)	38.1 (38.0–39.3)
Birth weight [kilograms]		3.1 (2.3–5.1)	3.0 (3.0–3.8)
Gender [males] (%)		12 (57)	4 (57)
Diagnosis [*n*] (%)	CDH	10 (48)	7 (100)
	MAS	5 (24)
	PPHN	5 (24)
	CCAM	1 (5)
Survival [*n*] (%)		18 (86)	7 (100)

Continuous data are presented as medians and range, discrete data as number and percentage. CDH: congenital diaphragmatic hernia, MAS: meconium aspiration syndrome, PPHN: persistent pulmonary hypertension of the neonate, CCAM: congenital cystic adenomatoid malformation.

**Table 2:** Macrocirculatory data.

	T1 ECMO	T2 ECMO	T1 Controls	T2 Controls	*P* value at baseline*	*P* value over time^†^
	*N* = 21	*N* = 21	*N* = 7	*N* = 7		
Age [days]	1 (0–12)	1 (0–12)	1 (0–6)	1 (0–7)	0.694	NA
Time to or from start ECMO [hours]	2 (0.5–24)	2 (0.5–24)	—	—	NA	NA
Time to or from ICU admission [hours]	2.5 (0.3–55.4)	6.4 (2.3–82.7)	12.4 (1.0–145.3)	33.5 (17.9–173.5)	NA	NA
Time between SDF measurements [hours]	4.0 (1.3–39.2)		26.8 (13.0–32.3)		0.005	
Heart rate [beats/min]	180 (120–220)	150 (106–198)	138 (113–191)	129 (110–160)	0.046	0.387
Mean blood pressure [mmHg]	49 (29–77)	49 (35–86)	44 (32–60)	52 (41–63)	0.264	0.727
Pulse pressure [mmHg]	19 (10–40)	10 (0–33)	25 (12–36)	24 (15–32)	0.559	<0.001
Vasopressor score	40 (0–140)	10 (0–108)	15 (0–75)	19 (0–66)	0.410	0.136
Dopamine [mcg/kg/min]	10 (0–20)	0 (0–20)	10 (0–21)	16 (0–21)	NA	NA
Dobutamine [mcg/kg/min]	10 (0–20)	5 (0–20)	10 (0–20)	5 (0–20)	NA	NA
Norepinephrine [mcg/kg/min]	0.1 (0.0–1.0)	0.0 (0.0–0.9)	0.0 (0.0–0.4)	0.0 (0.0–0.3)	NA	NA
Mean airway pressure [cm H_2_O]	18 (12–27)	11 (7–21)	14 (9–16)	13 (8–16)	0.019	0.357
Inhaled nitric oxide [ppm]	20 (0–40)	0 (0-0)	0 (0–19)	0 (0–20)	0.012	0.002
Oxygenation index	31 (5–94)	2 (1–21)	5 (3–13)	3 (0–7)	0.004	0.520
PELOD	20 (11–31)	—	11 (11–20)	—	0.006	—
Hemoglobin [mmol/L]	9.2 (6.9–12.6)	8.7 (6.7–12.0)	8.7 (7.4–11.0)	8.5 (7.8–10.8)	0.336	0.978
Hematocrit [L/L]	0.45 (0.32–0.62)	0.41 (0.31–0.56)	0.43 (0.38–0.53)	0.40 (0.34–0.53)	0.514	0.384
Fluid amount administered [mL/kg]	—	—	63 (10–145)	54 (26–112)	NA	NA
Fluid balance [mL/kg]	—	—	33 (6–139)	26 (−25–56)	NA	NA
Temperature [degrees Celsius]	37.4 (34.4–38.6)	36.9 (35.9–38.4)	37.3 (36.7–38.4)	36.8 (36.5–37.3)	NA	NA
ECMO flow [mL/kg/min]	—	140 (110–210)	—	—	NA	NA

Data are presented as median and range.

*Intergroup differences at T1 were assessed using Mann-Whitney test. ^†^For the time-dependent variables differences at T2 were assessed using ANCOVA with the baseline measurement as covariate.

NA: not assessed, —: not relevant, ECMO: extracorporeal membrane oxygenation, ICU: intensive Care Unit, PELOD: pediatric logistic organ dysfunction.

**Table 3:** Microcirculatory values.

	T1 ECMO	T2 ECMO	T1 Controls	T2 Controls	*P* value at baseline*	*P* value over time^†^
	*N* = 21	*N* = 21	*N* = 7	*N* = 7		
FCD [cm/cm^2^]	4.5 (2.4–7.7)	4.3 (2.8–7.4)	5.0 (1.8–7.2)	2.4 (1.4–4.2)	0.811	<0.001
MFI Large	2.76 (2.50–3.00)	2.88 (2.34–3.00)	2.92 (2.50–3.00)	3.00 (2.63–3.00)	0.266	0.367
MFI Medium	2.67 (2.13–3.00)	2.75 (2.13–3.00)	2.75 (2.38–3.00)	2.81 (2.50–3.00)	0.254	0.411
MFI Small	2.75 (2.06–3.00)	2.75 (2.08–3.00)	2.88 (2.44–3.00)	2.90 (2.63–3.00)	0.574	0.090
HI Large	0.10 (0.00–0.30)	0.09 (0.00–0.40)	0.09 (0.00–0.29)	0.00 (0.00–0.26)	0.951	0.2406
HI Medium	0.14 (0.00–0.60)	0.11 (0.00–0.35)	0.10 (0.00–0.51)	0.00 (0.00–0.27)	0.736	0.2421
HI Small	0.18 (0.00–0.73)	0.09 (0.00–0.37)	0.09 (0.00–0.40)	0.00 (0.00–0.17)	0.579	0.0971

Data are presented as median and range.

*Intergroup differences at T1 were assessed using Mann-Whitney test. ^†^For the time-dependent variables, differences at T2 were assessed using ANCOVA with the baseline measurement as covariate.

FCD: functional capillary density, MFI: microvascular flow index, HI: heterogeneity index.
